# Implementation of Q learning and deep Q network for controlling a self balancing robot model

**DOI:** 10.1186/s40638-018-0091-9

**Published:** 2018-12-21

**Authors:** MD Muhaimin Rahman, S. M. Hasanur Rashid, M. M. Hossain

**Affiliations:** 1 Department of Mechanical Engineering, Bangladesh University of Engineering and Technology, Dhaka, Bangladesh; 2 Department of Electrical and Electronic Engineering, Bangladesh University of Engineering and Technology, Dhaka, Bangladesh

## Abstract

In this paper, the implementations of two reinforcement learnings namely, Q learning and deep Q network (DQN) on the Gazebo model of a self balancing robot have been discussed. The goal of the experiments is to make the robot model learn the best actions for staying balanced in an environment. The more time it can remain within a specified limit, the more reward it accumulates and hence more balanced it is. We did various tests with many hyperparameters and demonstrated the performance curves.

**Electronic supplementary material:**

The online version of this article (10.1186/s40638-018-0091-9) contains supplementary material, which is available to authorized users.

## Introduction

Control system is one of the most critical aspects of Robotics Research. The Gazebo is one of the most robust multi-robot simulators at present. The ability to use the Robot Operating System (ROS) with Gazebo makes it more powerful. However, there is very few documentation on how to use ROS and Gazebo for Controllers development. In our previous paper, [1], we attempted to demonstrate and document the use of PID, Fuzzy logic and LQR controllers using ROS and Gazebo on a self-balancing robot model. Later on, we have worked on Reinforcement learning. In this paper, we have the implementation of Q Learning and Deep Q Network on the same model. The paper is structured as follows. “Related works” section shows the related works on the subject. “Robot model” section discusses the Robot Model. “Reinforcement learning methods as controllers” section shows the implementation of Q Learning and DQN as controllers. Finally, “Conclusion and future work” section is the conclusion.

## Related works

Lei Tai and Ming Liu [2] had worked on Mobile Robots Exploration using CNN based reinforcement learning. They trained and simulated a TurtleBot on Gazebo to develop an exploration strategy based on raw sensor value from the RGB-D sensor. The company *ErleRobotics* have extended OpenAI environment to Gazebo [3]. They have deployed Q-learning and Sarsa algorithms for various exploratory environments. Loc Tran et al. [4] developed a training model for an Unmanned aerial vehicle to explore with static obstacles in both Gazebo and the real world, but their proposed Reinforcement learning is unclear from the paper. Volodymyr Sereda [5] used Q-learning on a custom Gazebo model using ROS in exploration strategy. Rowan Border [6] used Q-learning with neural network presentation for robot search and rescue using ROS and Turtlebot.

## Robot model

**Fig. 1:**
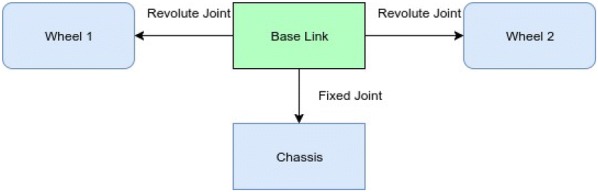
Simple block diagram of the model

**Fig. 2:**
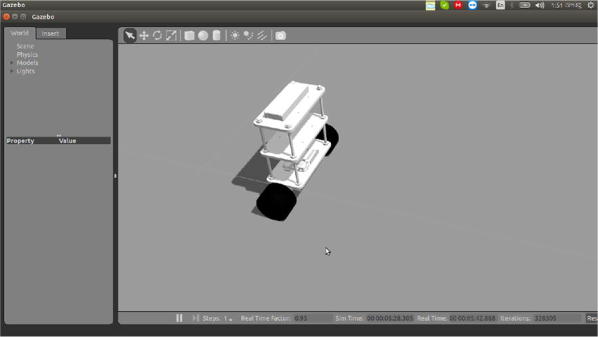
Gazebo model

The robot model is described in the paper [1]. It has one chassis and two wheels. The task of the model is to keep the robot balanced, i.e., keeping its pitch angle in between ± 5°. The more it remains in between the limits, the more it gets the reward. Figure 1 shows the block diagram and Fig. 2 shows the Gazebo model of the self-balancing robot.

### Controller

**Fig. 3:**
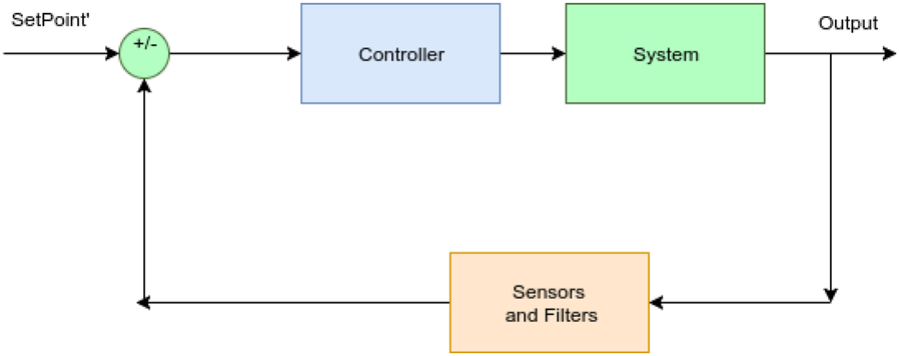
Controller block diagram

The robot’s IMU sensor measures the roll, pitch and yaw angles of the chassis every second and sends them to the controller. The controller then calculates optimum action value to make the chassis tilt according to set point. Figure 3 shows the control system of the robot.

## Reinforcement learning methods as controllers

Previously, we worked on traditional Controllers like PID, Fuzzy PD, PD+I & LQR [1]. The biggest problem with those methods is that they need to be tuned manually. So, reaching optimal values of controllers depends on many trials and errors. Many times optimum values aren’t achieved at all. The biggest benefit of reinforcement learning algorithms as controllers is that the model tunes itself to reach the optimum values. The following two sections discuss Q Learning and Deep Q Network (Additional file 1).

### Q learning

Q-learning was developed by Christopher John Cornish Hellaby Watkins [7]. According to Watkins, “it provides agents with the capability of learning to act optimally in Markovian domains by experiencing the consequences of actions, without requiring them to build maps of the domains” [8]. In a Markovian domain, *Q* function—the model to be generated using the algorithm—calculates the expected utility for a given finite state *s* and every possible finite action *a*. The agent—which is the robot in this case—selects the optimum action *a* having the highest value of *Q*(*s*, *a*) , this action choosing rule is also called Policy [8]. Initially, the *Q*(*s*, *a*) function values are assumed to be zero. After every training step, the values get updated according to the following equation (Additional file 2)

1 $$\begin{aligned} Q(s,a_t) \leftarrow Q(s,a_t)+ \alpha (r+\gamma maxQ(s_{t+1},a)) \end{aligned}$$

#### Algorithm

The objective of the model in our project is to keep it within limits, i.e., ± 5°. At first, the robot model, *Q* matrix, policy $\pi$ are initialized. There are some interesting points to make. The states are not finite. Within the limit range, hundreds and thousands of pitch angles are possible. Having thousands of columns is not possible. So, we discretized the state values into 20 state angles from − 10° to 10°. For action value, we chose ten different velocities and they are [− 200, − 100, − 50, − 25, − 10, 10, 25, 50, 100, 200] ms^−1^. The *Q* matrix had 20 columns, each column representing a state and ten rows each representing every action. Initially, the *Q*-values were assumed to be 0, and some random actions were specified for every state in the policy $\pi$. We trained for 1500 episodes, each episode having 2000 iterations. At the beginning of each episode, the simulation refreshed. Whenever the robot’s state exceeded the limit, it was penalized by assigning a reward to $-100$. The *Q* Table is updated at each step according to Eq. 1. The Algorithm 1 shows the full algorithm. (Additional file 3)

#### Result and discussion

**Fig. 4:**
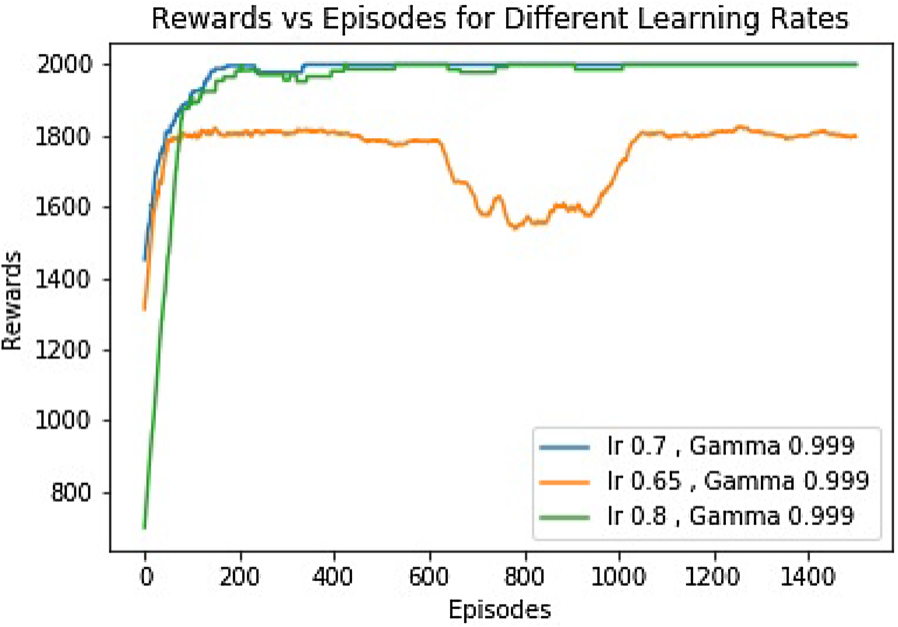
Rewards for different $\alpha$


The simulation was run for three different $\alpha$ values (0.7, 0.65, 0.8), with $\gamma$ value of (0.999). Figure 4 shows the rewards vs episodes for those $\alpha$s. It is evident that the robot couldn’t earn the targeted amount of rewards within the training period for those learning rates. We see that, for the $\alpha$ values of 0.7 and 0.8, the robot reached at maximum possible accumulated rewards, 2000, within 400 episodes. The curve with the $\alpha$ value of 0.7 is less stable compared to that of 0.8. However, The curve with the $\alpha$ value of 0.65 never achieved the maximum accumulated reward (Additional file 4).

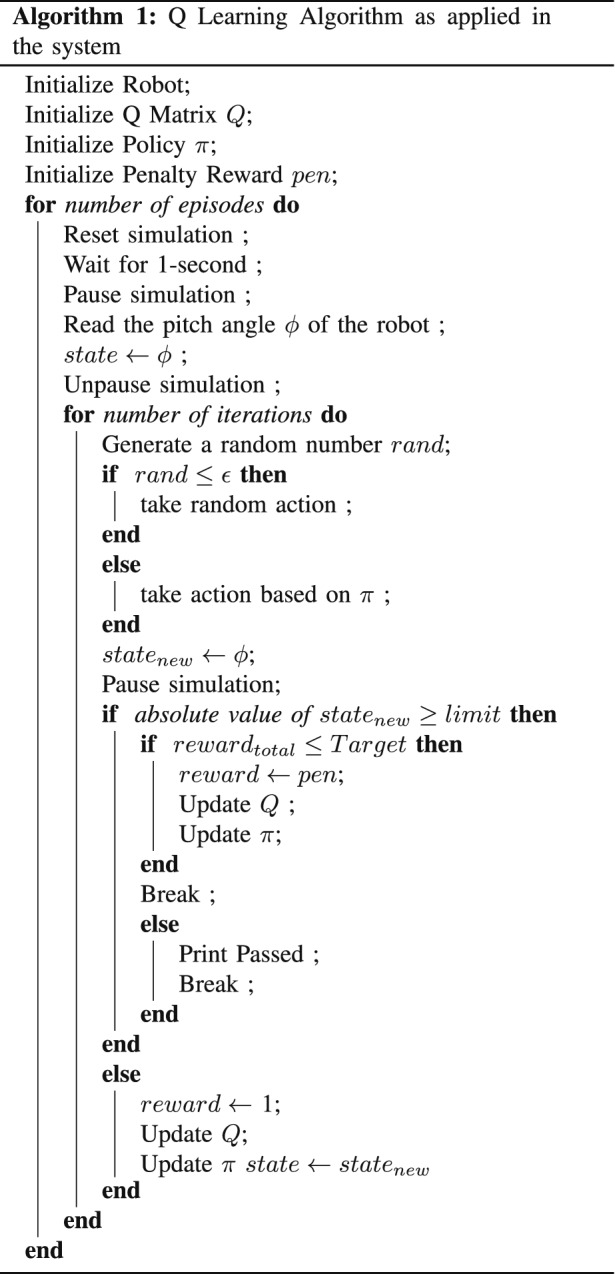


### Deep Q network (DQN)

Mnih et al. [9] first used Deep Learning as a variant of Q Learning algorithm to play six games of Atari 2600, which outperformed all other previous algorithms. In their paper, two unique approaches were used.

- Experience Replay
- Derivation of Q Values in one forward pass (Additional file 5).

#### Experience replay

The technique of Experience Replay, experiences of an agent, i.e., $(state, reward, action,state_{new})$ are stored over many episodes. In the learning period, after each episode, random batches of data from experience are used to update the model [9]. According to the paper, there are several benefits to such an approach (Additional file 6). They are-

- It allows greater data efficiency as each step of experience can be used in many weight updates
- Randomizing batches break correlations between samples
- Behaviour distribution is averaged over many of its previous states.

#### Derivation of Q values in one forward pass

**Fig. 5:**
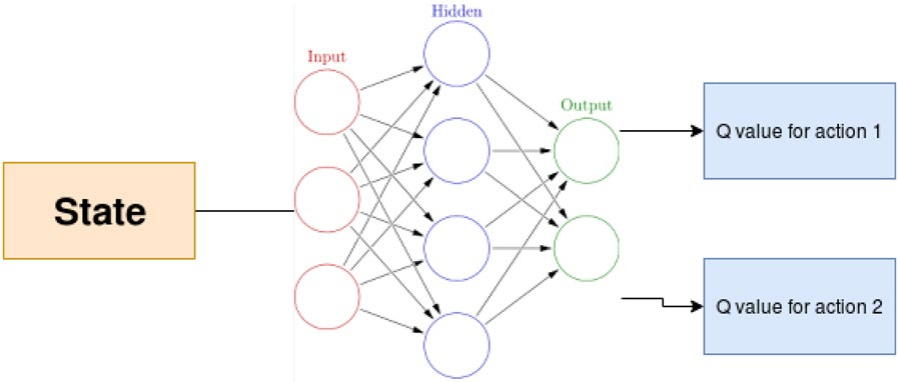
Sample deep Q network architecture

In the classical Q learning approach, one has to give state and action as an input resulting in *Q* value for that state and action. Replicating this approach in Neural Network is problematic as one has to give state and action for each possible action of the agent to the Model (Additional file 7). It will lead to many forward passes in the same model. Instead, they designed the model in such a way that it will predict Q values for each action for a given state. As a result, only one forward pass is required. Figure 5 shows a sample architecture for one state with two actions

#### Implementation on the robot model

The implementation of the DQN on our Robot model is similar to *Q* Learning Method. However, there are some exceptions. At first, a model was initialized instead of Initializing *Q* matrix. In the $\epsilon$ greedy policy, instead of choosing the action based on policy $\pi$, *Q* values were calculated according to the model. At the end of every episode, the model was trained using random mini batches of experience. At first, an architecture with two hidden Relu layers of 20 units was selected whereas the last layer was a Linear Dense layer with ten units. With the $\gamma$ of 0.999 and $\alpha$ of (0.65, 0.7, 0.8) . Algorithm 2 shows the DQN algorithm as implemented on the robot model.
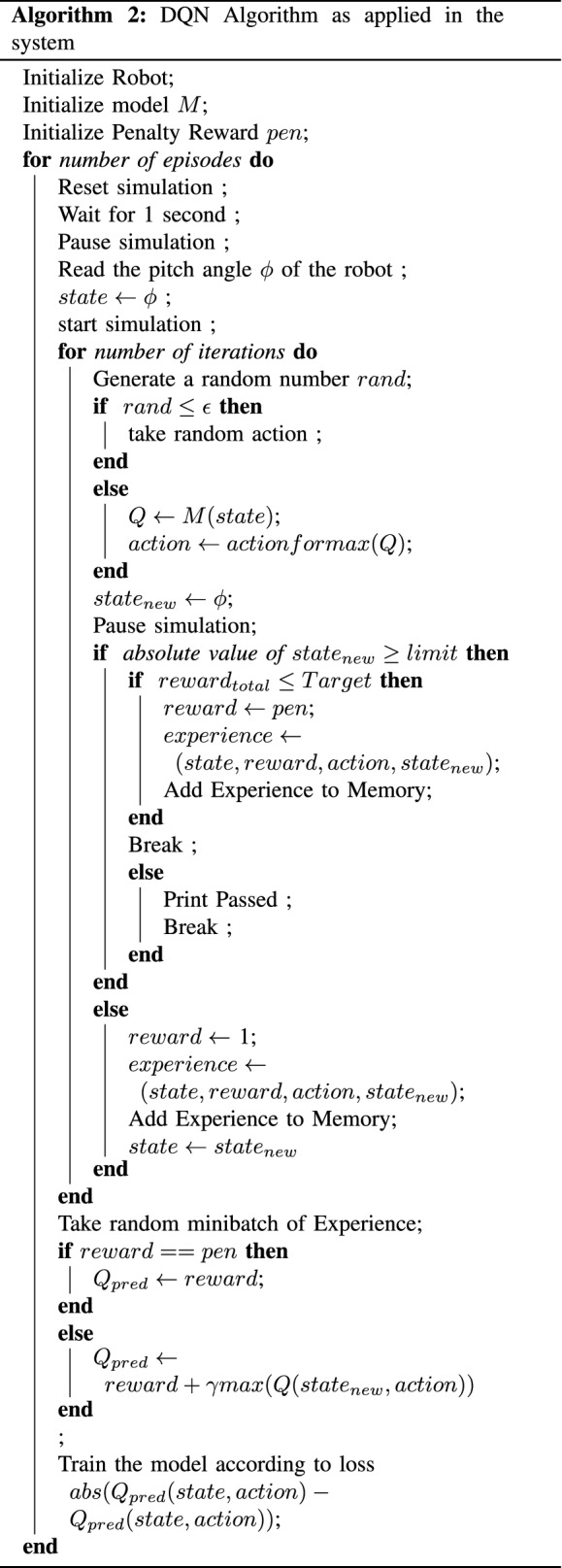


#### Architecture

**Fig. 6:**
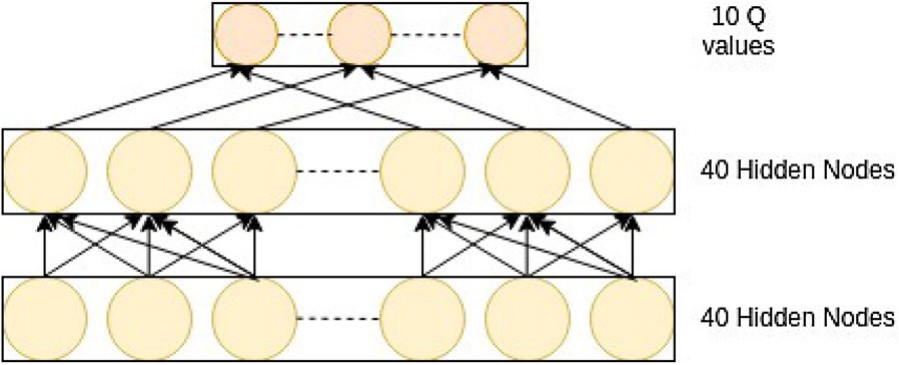
Schematic diagram of DQN architecture used

The architecture of the model is simple. It is a Multi-layer perceptron network, with two hidden layers of 40 nodes. the last layer is of 10 output nodes. The activation function we used in every hidden layer is Rectified Linear Unit. The last layer has linear activation function (Fig. 6).

#### Training

**Fig. 7:**
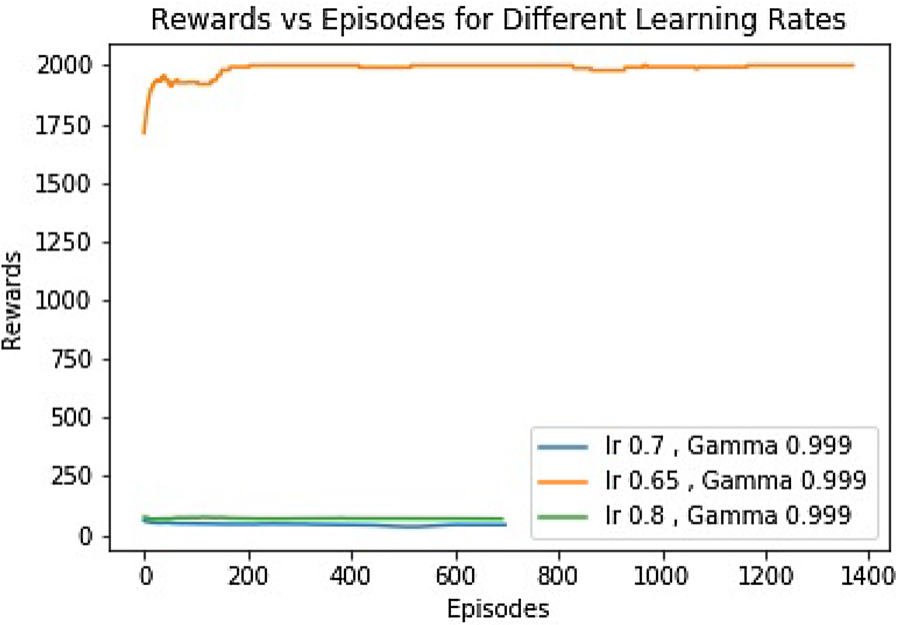
Rewards for three different $\alpha$s with $\gamma$ 0.999

**Fig. 8:**
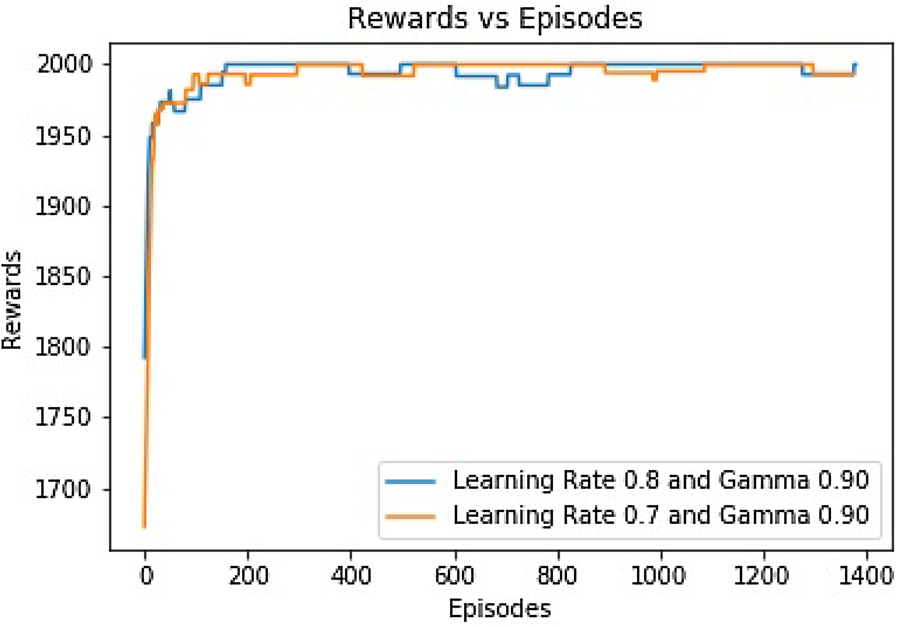
Rewards versus episodes for new architecture

From Fig. 7, we see that the total rewards for $\alpha$ (0.65) are significantly higher. It starts approximately from 1750 and reaches the maximum total rewards, 2000 within the 200th episode. However, the accumulated rewards with $\alpha$ values of 0.7and 0.8 are meager. They have accrued rewards approximately 50–60 for the whole time. Later, the architecture was changed to 2 hidden layers of 40 Relu Units where the value of $\gamma$ was selected to be 0.9. Figure 8 shows that both curves reached the highest accumulated rewards within 200 episodes in the new configuration.

**Fig. 9:**
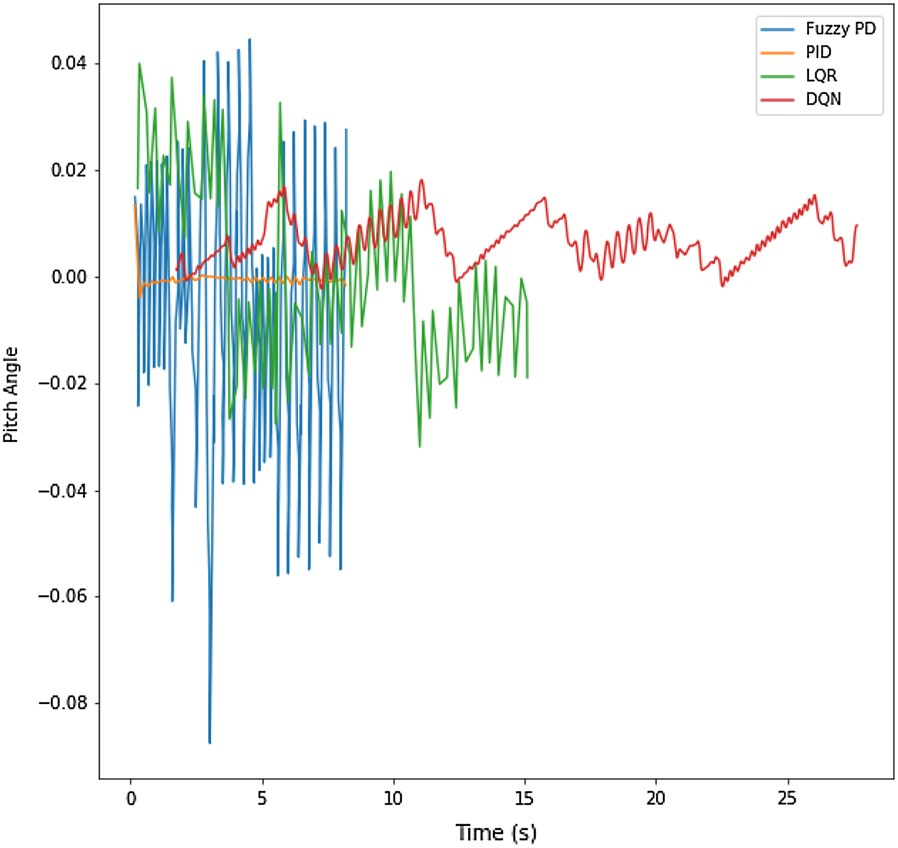
Performance curve for PID, fuzzy logic and LQR

## Comparison to traditional methods

In our previous paper, [1], we evaluated the performance of PID, Fuzzy Logic and LQR on a self-balancing robot model and compared among those controllers. Figure 9 shows the performance curves for PID, Fuzzy P, LQR and DQN. It shows that LQR and Fuzzy controllers were not so stable like PID, although we had to tune all of them manually. The DQN performance curves are more stable than fuzzy P and LQR.But less stable than PID. There are two reasons behind being less stable can be, that the PID algorithm is giving continuous action values, while our architecture is designed for discrete values. The second reason is the reward function for this architecture is to limit the pitch angle between − 5° and 5°. Narrowing down that range will help the architecture to perform better (Additional file 8).

## Conclusion and future work

The implementation of *Q* Learning and Deep *Q* Network as a controller in the Gazebo Robot Model was shown in this paper. It showed the details of the algorithms. However, some further improvements can be made. Like, It was assumed that the robot would work on Markovian State space, which generally not the case. In general, Inverted pendulum models are Non-markovian models. So there must exist some dependencies among the states. So In future, Recurrent Neural Network has a great possibility. Moreover, ten predefined values of velocities for action were used. In the real world application, action values have continuous range. So for more complex models, this method may not work. In that case, deep reinforcement learning algorithms with continuous action space like Actor-Critic Reinforcement Learning algorithm [10] can be used. Finally, this work should be improved toward real-world scenarios.

## Additional file

**Additional file 1.** PID1:Performance Values of PID with Kp 100, Ki 0.5, Kd 0.1.

**Additional file 2.** FuzzyPD: Performance Values of Fuzzy PD control system.

**Additional file 3.** FuzzyPD+I: Performance Values of Fuzzy PD+ I control system.

**Additional file 4.** LQ1R1: Performance values of LQR control system with Q 10 and R 100.

**Additional file 5.** LQ2R2: Performance values of LQR control system with Q 100 and R 1000.

**Additional file 6.** PID2: Performance values of PID control system Kp 50, Ki 0.8 and kd 0.05.

**Additional file 7.** PID3: Performance values of PID control system Kp 25, Ki 0.8 and kd 0.1.

**Additional file 8.** P1: Performance values of P control system Kp 50000.
